# Giant second-harmonic generation in monolayer MoS_2_ boosted by dual bound states in the continuum

**DOI:** 10.1515/nanoph-2024-0273

**Published:** 2024-07-11

**Authors:** Ji Tong Wang, Jian Wei You, Nicolae C. Panoiu

**Affiliations:** University College London, London, UK; Southeast University, Nanjing, China

**Keywords:** dielectric metasurfaces, bound states in the continuum, monolayer molybdenum disulfide, second-harmonic generation, eigenmode expansion method, nonlinear homogenization

## Abstract

Dielectric metasurfaces open new avenues in nonlinear optics through their remarkable capability of boosting frequency conversion efficiency of nonlinear optical interactions. Here, a metasurface consisting of a square array of cruciform-shaped silicon building blocks covered by a monolayer MoS_2_ is proposed. By designing the metasurface so that it supports optical bound states in the continuum (BICs) at the fundamental frequency and second harmonic, nearly 600× enhancement of the second-harmonic generation (SHG) in the MoS_2_ monolayer as compared to that of the same MoS_2_ monolayer suspended in air is achieved. To gain deeper insights into the physics of the metasurface-induced enhancement of nonlinear optical interactions, an eigenmode expansion method is employed to analytically investigate the main characteristics of SHG and the results show a good agreement with the results obtained *via* full-wave numerical simulations. In addition, a versatile nonlinear homogenization approach is used to highlight and understand the interplay between the BICs of the metasurface and the efficiency of the SHG process. This work suggests a promising method to enhance the nonlinear optical processes in two-dimensional materials, enabling the development of advanced photonic nanodevices.

## 1 Introduction

Optical metasurfaces, which consist of artificial, ultra-thin periodic nanostructures, have attracted rapidly growing research interest in recent years, chiefly due to their remarkable versatility in light manipulation, including phase control, distribution of the optical near-field, and light polarization. Compared to traditional optical bulk materials, two-dimensional (2D) metasurfaces exhibit appealing advantages derived from their large design parameter space and the availability of diverse, versatile nanofabrication techniques. These properties of metasurfaces have spurred rapid technological advancements in integrated optics, sensing, information processing, and many other areas [1]–[3]. Notably, the ability of metasurfaces to produce significant enhancement of the local optical field makes them a prominent and promising platform for nonlinear optics. This has resulted in the emergence of nonlinear metasurfaces as key tools for advancing our understanding of nonlinear optical phenomena at the nanoscale [4]–[6], with a central practical goal being a significant improvement of the optical power conversion efficiency of nonlinear optical processes. In this context, to further boost the efficiency of frequency conversion processes, it is of critical importance to find materials with large optical nonlinearity and to develop innovative approaches that enable improved light–matter coupling at deep-subwavelength scale.

The discovery of monolayer semiconducting 2D materials, including transition metal dichalcogenides (TMDs), has provided a fertile ground for the development of fundamental new ideas and device applications in optoelectronics and photonics [7]–[9], chiefly because of their unique optical properties stemming from reduced dimensionality. In near-infrared and visible spectral domains, direct bandgaps of monolayer TMDs [10], [11] permit much stronger light emission as compared to their bulk counterparts, and their intense excitonic effects [12], [13] enable novel applications based on optical devices operating at room-temperature. Moreover, strong nonlinear optical response of monolayer TMDs has been observed recently [14]–[18], including nonlinear edge states and second-harmonic generation (SHG). The non-centrosymmetric nature of the atomic structure of monolayer TMDs allows for a broader set of nonlinear optical interactions, which in turn offers increased versatility in the design of novel active photonic nanodevices. On the other hand, the 2D nature of TMD materials inherently limits the efficiency of light–matter interactions, especially in the nonlinear optical regime [19]. Therefore, there is a pressing demand for designing feasible approaches to increase the light coupling to monolayer TMDs. In this context, nonlinear metasurfaces provide one of the most promising routes towards achieving this critical goal.

Generally, optical metasurfaces can be divided into two classes, namely metallic metasurfaces [20]–[22] relying on plasmonic resonances and dielectric metasurfaces [23]–[25] using dielectric materials with high index of refraction. Plasmonic metasurfaces display remarkably strong resonances but generally exhibit large optical losses. By contrast, dielectric metasurfaces have low optical losses but the field enhancement they induce is rather modest. Recently, a novel field-enhancement mechanism based on bound states in the continuum (BICs) [26]–[28] has been introduced, and its effectiveness in enhancing the optical coupling between light and periodically structured metasurfaces has been demonstrated. In principle, optical BICs cannot couple to free-space radiative modes, a property that implies a practically infinite quality-(*Q*) factor and vanishingly small line-width. However, in practice, due to roughness and other inherent fabrication imperfections, ideal BICs transition to resonances with finite *Q*-factor called quasi-BICs [29]–[31]. Despite this, it has recently been shown that one can design dielectric metasurfaces with broken in-plane inversion symmetry [32], [33], which can possess quasi-BICs with particularly large *Q*-factors. In nonlinear optics, BICs provide a novel approach to generate a large enhancement of nonlinear optical interactions [34]–[36], mainly *via* significant localization and enhancement of the optical near-field. Whereas the enhancement of nonlinear optical interactions in 2D materials due to the excitation of a BIC has been addressed [18], [37], the highly nontrivial case in which BICs exist at multiple frequencies has yet to be investigated.

In this paper, we propose a novel approach to enhance SHG in 2D TMD materials whereby the optical fields at the fundamental-frequency (FF) and second-harmonic (SH) that interact with the 2D material represent BICs of a specially engineered silicon metasurface. More specifically, the nonlinear metasurface consists of a square array of cruciform-shaped silicon nanoparticles on top of which a monolayer molybdenum disulfide (MoS_2_) is deposited. By finely tuning the asymmetry of the silicon crosses, one can design a metasurface that possesses high-*Q* optical quasi-BICs at FF (*ω*) and SH (Ω = 2*ω*). In this configuration, the SHG in a MoS_2_ layer placed on top of silicon crosses is nearly three orders of magnitude larger than that corresponding to a suspended MoS_2_ layer. To gain deeper physical insights into this BIC-based mechanism for enhancement of SHG in 2D materials, a theoretical analysis of the enhancement of the SHG based on the eigenmode expansion method is developed and used to estimate the optical power generated at the SH. The corresponding conclusions are validated by the results obtained using rigorous numerical simulations. Finally, we use a versatile homogenization method to calculate the effective second-order susceptibility of monolayer MoS_2_, which can be subsequently employed to quantify the enhancement of the SHG due to the presence of BICs at both the FF and SH.

## 2 Results and discussion

In this section, we present and discuss the main results of our study. We begin by summarizing the properties of the linear optical response of our nonlinear metasurface, the focus being on identifying the system resonances. We then analyze the nonlinear optical response of the metasurface using both a theoretical model based on modal expansion method as well as rigorous numerical simulations. We conclude this section with the presentation of a nonlinear homogenization method that allows us to readily calculate the effective nonlinear second-order susceptibility of the silicon/MoS_2_ composite metasurface and subsequently the spectral location of system resonances.

### 2.1 Geometrical configuration

**Figure 1:**
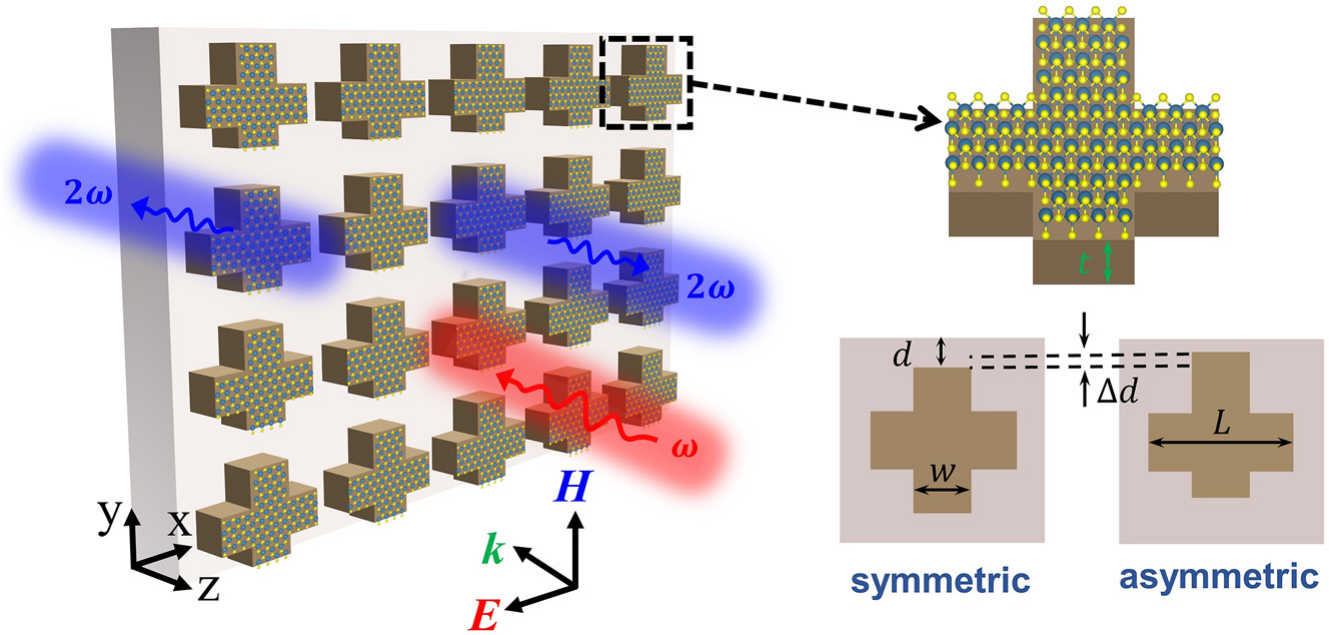
Schematics of a nonlinear metasurface with monolayer MoS_2_ on top of its cruciform-shaped silicon unit cell. Asymmetry parameter is defined as *s* = Δ*d*/*d* and the proposed metasurface is illuminated by a normally incident, *x*-polarized plane wave.

The proposed nonlinear metasurface is schematically illustrated in Figure 1. It consists of a periodic square array whose unit cell consists of a cruciform silicon nanoparticle fully covered by a monolayer MoS_2_ with identical cross-section. There are a few reasons why we chose in this work cruciform-shaped nanoparticles made of silicon. First, the optical properties of this class of nanoparticles have been thoroughly investigated in the past, and therefore it provides a convenient way to validate some of our results. Second, cruciform-shaped nanoparticles can be easily fabricated as they have a simple geometrical configuration. Third, silicon is one of the most widely used optical materials in nanophotonics, so that it is a natural material choice. Finally, the broken in-plane inversion symmetry of the optical metasurface can be easily implemented and quantified using the cruciform configuration.

The array is placed on a quartz substrate. The period of the unit cell along both *x*- and *y*-axes is *a* = 506 nm. The length and width of each arm of the crosses are *L* = 316 nm and *w* = 126 nm, respectively, whereas their thickness is *t* = 122 nm. The monolayer MoS_2_ completely covers the silicon nanoparticles and its thickness is 0.615 nm [38]. In the case of symmetric cruciforms, the centers of the two arms of the cruciform coincide with the center of the unit cell.

To create the conditions necessary for the existence of quasi-BICs, we shift the cruciform arm aligned with the *y*-axis so as to break the in-plane inversion symmetry (see the upper right corner of Figure 1). From geometrical considerations, one can infer that the maximum distance by which the arm can be shifted along the *y*-axis is *d* = 95 nm. To quantify the degree of asymmetry of the crosses we introduce an asymmetry parameter defined as *s* = Δ*d*/*d*, which in principle can vary from 0 to 1. Moreover, by shifting the arm of the cross one breaks the in-plane inversion symmetry of the optical system and, as a result, the symmetry-protected BIC mode becomes a quasi-BIC mode. This composite nonlinear metasurface is illuminated by a normally incident, *x*-polarized plane wave with frequency, *ω*. Further details pertaining to the linear and nonlinear optical properties of silicon and MoS_2_ are given in the Supplementary Material.

### 2.2 Linear optical response of the silicon/MoS_2_ composite metasurface

In this section, we investigate the linear optical response of the proposed nonlinear metasurface. To seek for BICs of the metasurface and investigate the relationship between the quasi-BIC modes and the asymmetry parameter *s*, we first determine the dependence of the band structure and frequency dispersion properties of the resonant modes of the proposed nonlinear metasurface on the asymmetry parameter. Details about this computational procedure and all other computational methods used in this work are provided in Section 1 of the Supplementary Material. In addition, to design a nonlinear metasurface possessing the dual-resonance feature, namely a metasurface that supports quasi-BICs at the FF and SH, we also study the spectral characteristics of the band structure of the metasurface in the vicinity of SH frequencies.

**Figure 2:**
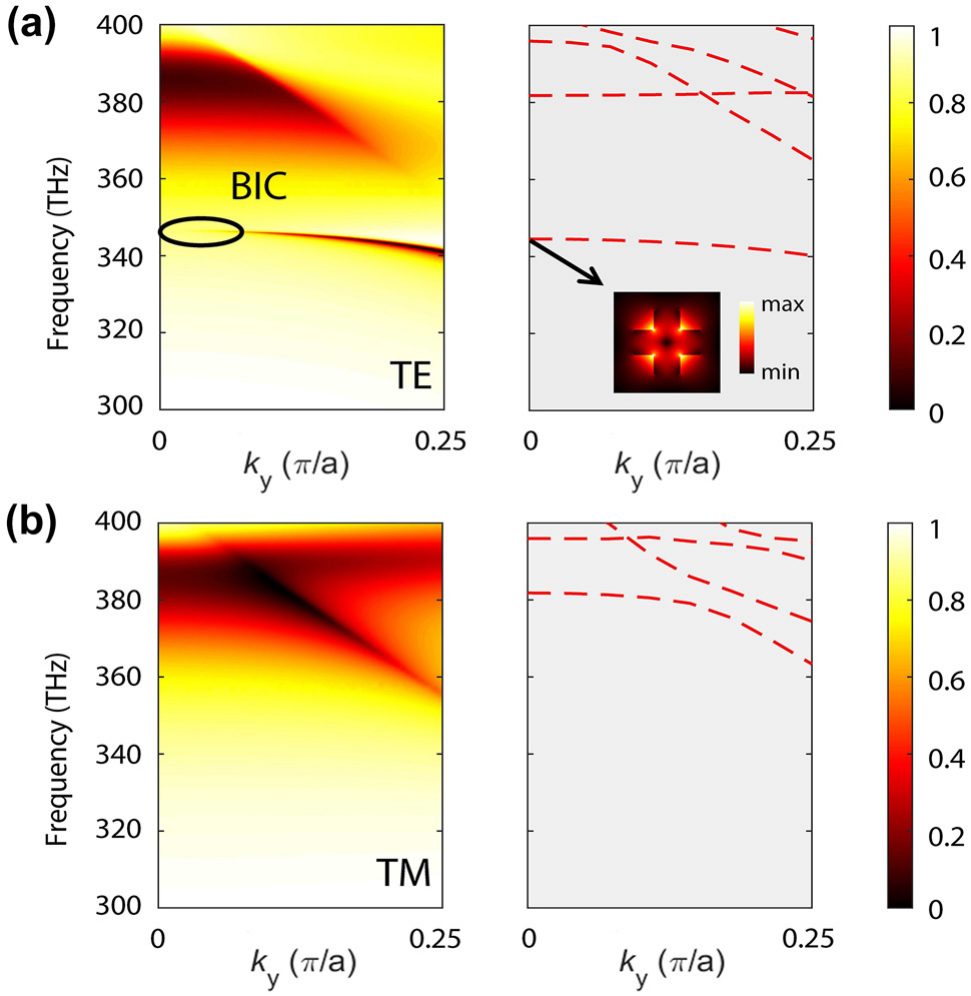
Dispersion maps and band diagrams calculated for *s* = 0 at the FF. (a) Dispersion map of transmission spectra (left panel) corresponding to TE-polarized incident plane waves. The symmetry-protected BIC is marked with a black ellipse. The right panel shows the TE-like eigenmodes depicted on a grey background denoting the radiation continuum. The inset shows the distribution of the normalized electric field in the *x*–*y* cross-section of the unit cell of the BIC mode at the Γ-point. (b) Same as in (a), but determined for TM-polarized incident plane waves.

To characterize the spectral optical response of our nonlinear metasurface, we determined the angle-resolved dispersion map of the transmission and band diagram of the optical modes corresponding to the asymmetry parameter *s* = 0. The results of these calculations are summarized in Figure 2, where the incident angle in transmission map has been converted to the wavevector *k*_*y*_ (in our calculations, $k_{y}\in \left[0,0.25\pi /a\right]$ ), so that one can easily identify the bands in the transmission map by comparing with those in the band diagram determined within the same wavevector range. We performed these calculations for both transverse electric (TE)-like and transverse magnetic (TM)-like modes.

By comparing the left and right panels in Figure 2a and B, one can see that the corresponding band structures are consistent with the frequency dispersion of resonances in the transmission maps. Moreover, the left panel of Figure 2a shows a BIC with frequency of 345.8 THz located at the Γ-point (corresponding to an angle of incidence equal to zero). The in-plane spatial distribution of the electric field of the BIC mode at the Γ-point is depicted in the inset of the right panel of Figure 2a. The linewidth of the BIC resonance vanishes at the Γ-point, because of the symmetry incompatibility between the BIC and radiative modes, and increases gradually away from the Γ-point, reflecting its transition to a quasi-BIC. Furthermore, the BIC band in Figure 2a proves that away from the Γ-point incoming light can couple to the nonlinear metasurface. In the case of the TM polarization (Figure 2b), there are no BICs within the frequency range of interest. Thus, this analysis confirms that the proposed silicon metasurface can support a symmetry-protected BIC at the Γ-point under its excitation with TE-polarized waves.

To facilitate the optical coupling to the nonlinear metasurface, an asymmetric perturbation should be introduced to break the symmetry of the unit cell and transform the BIC mode to a quasi-BIC mode. In previous studies [32], [33], [39], the asymmetric perturbation was introduced by removing part of the elements forming the unit cell. Here, we use a more convenient approach to break the inversion symmetry, namely we shift one arm of the cruciform silicon resonator. The advantage of this approach is that the total area of the cruciform MoS_2_ patch remains unchanged when we vary the asymmetry parameter. This invariance of the area of the MoS_2_ layer where SH is generated makes it particularly convenient to quantitatively compare the enhancement factor of nonlinear optical response of metasurfaces with different values of *s*.

**Figure 3:**
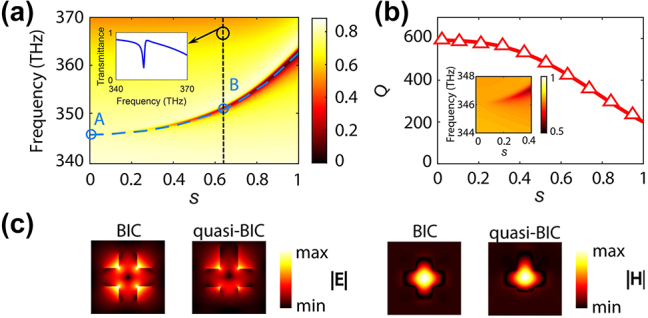
Characterization of the BIC mode. (a) Dispersion map of the transmission spectra, determined within the range of the FF for varying values of the asymmetry parameter, *s*. The blue dashed line indicates the resonance of the quasi-BIC mode, and the modes labeled by *A* and *B* correspond to the BIC (*s* = 0) and a quasi-BIC with *s* = 0.63, respectively. The inset shows the transmission spectrum at *s* = 0.63. (b) Dependence of *Q*-factor of quasi-BIC mode on *s*. In inset, a zoom-in of the dispersion map of the transmission spectra shows the vanishingly small transmission at *s* = 0. (c) In-plane spatial distribution of the electric and magnetic fields corresponding to the modes labeled by *A* and *B* in (a).

By breaking the in-plane inversion symmetry of the metasurface, the symmetry-protected BIC will be converted to a quasi-BIC, which can be used to dramatically enhance the light–matter interaction. To illustrate the influence of the asymmetry parameter on the quasi-BIC mode, we vary *s* from 0 to 1 and calculate the transmission for normally incident plane waves linearly polarized along the *x*-axis. As indicated in Figure 3a, the BIC mode corresponding to *s* = 0 has frequency 345.8 THz, and the frequency of the quasi-BIC mode increases as the asymmetry parameter increases.

The signature of the quasi-BIC mode is imprinted in the transmission spectra as a sharp and asymmetric resonance with Fano-line shape [32] (see Figure 3a). To understand how the variation of the asymmetry parameter affects the properties of the quasi-BIC, we extracted the *Q*-factor of the corresponding resonance by fitting the transmission spectrum with the Fano formula given by $T_{Fano}={\left|a_{1}+ia_{2}+\frac{b}{\omega -\omega_{0}+i\gamma }\right|}^{2}$ , where *a*_1_, *a*_2_, and *b* are constant coefficients, *ω*_0_ is the resonance frequency, and *γ* is the leakage rate of the resonance. Then, the *Q*-factor can be determined from the relation *Q* = *ω*_0_/2*γ* [23]. As suggested by Figure 3b, the *Q*-factor increases when *s* decreases, reaching a value of about 600 when *s* = 0. More specifically, when *s* = 0, which corresponds to the BIC, the Fano parameters become ill-defined since the resonance peak in the transmission spectrum vanishes – see the inset in Figure 3b. The fact that the *Q*-factor of the BIC is finite is explained by the intrinsic optical absorption in silicon. In the lossless case, the value of the *Q*-factor of the BIC would be infinite.

For illustration, we present in Figure 3c the spatial distribution of the electric and magnetic fields corresponding to the BIC mode (*s* = 0) and a generic BIC-like mode (*s* = 0.63). For *s* = 0 (point *A* in Figure 3a) the electric and magnetic field profiles of the BIC mode of the silicon metasurface are invariant upon in-plane inversion symmetry transformation, leading to complete decoupling between the BIC mode and the incident plane wave. Unlike the case of the BIC resonance, the field profiles corresponding to the quasi-BIC mode lack in-plane symmetry.

In addition to the FF regime, we also studied the linear optical response of the proposed metasurface within the frequency range where the SH is generated. To investigate the enhancement of the nonlinear optical response of the metasurface through the double-resonance effect, we determined the angle-resolved transmission maps and looked for optical resonances in the spectral domain where SH would be generated. In order to achieve a strong nonlinear optical interaction, one needs to optimize the overlap of the optical near-fields of the modes at the FF and SH. Given the symmetry properties of the TE-like, quasi-BIC mode at the FF and those of the second-order nonlinear susceptibility tensor, ***χ*** ^(2)^(**r**; Ω, *ω*, *ω*), of single-layer MoS_2_ (for details see Section 2 of the Supplementary Material), the generated nonlinear polarization **P**_*nl*_(**r**, Ω) = *ϵ*_0_ ***χ*** ^(2)^(**r**; Ω, *ω*, *ω*): **E**(**r**, *ω*)**E**(**r**, *ω*) is antisymmetric about the reflection plane *x* = 0 and only couples with TE-like modes at SH. This idea plays a central role in our theoretical analysis and numerical simulations.

**Figure 4:**
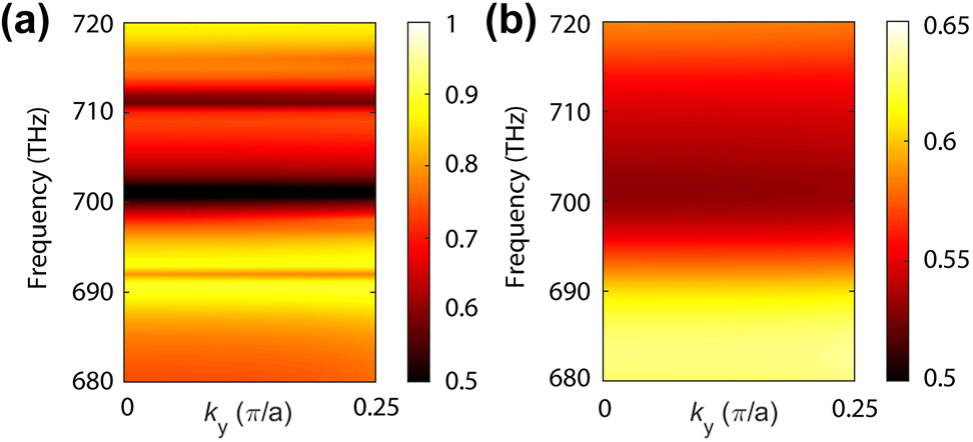
Dispersion maps at the SH for symmetric metasurfaces (*s* = 0). (a), (b) Angle-resolved transmission map at SH frequencies calculated in the cases when optical losses are neglected and when they are taken into account, respectively. A TE-polarized plane wave is used and the asymmetry parameter *s* = 0.

To focus on the relevant physics, we first neglected the optical losses in silicon, the corresponding angle-resolved transmission map determined for *s* = 0 being presented in Figure 4a. We can clearly observe in this figure several resonances whose frequency hardly varies with the wavevector. As can be seen in Figure 4b, when the optical losses in silicon are incorporated in our calculations, the resonances broaden and merge together, resulting in insignificant resonant features within the studied frequency spectrum. In spite of this, the existence of a few resonances (Figure 4a) in the vicinity of SH frequency implies that it is possible to design the parameters of the silicon metasurface in such a way that it possesses a quasi-BIC mode at the FF (around 350 THz) and another quasi-BIC at the SH (around 700 THz). Later on, we will quantify the enhancement of the SHG induced by this double-resonance phenomenon.

**Figure 5:**
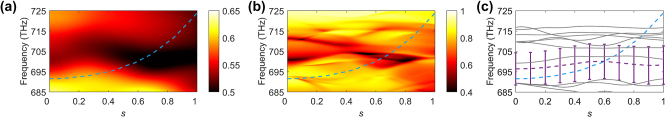
Symmetry-dependent dispersion maps and double-resonance phenomenon. (a) Dispersion map of transmission determined in the spectral range of SH and with optical losses included. The metasurface is excited by a normally incident plane wave polarized along the *x*-axis. (b) Same as in (a), but in the lossless case. (c) Frequency bands of TE-like modes versus *s*, with optical losses included. The purple dashed line depicts the optical mode dominant at the SH. The error bars denote the linewidth. In these plots, the blue dash lines are the quasi-BIC resonance at FF *ω*_0_ plotted in terms of 2*ω*_0_.

One convenient approach to illustrate the procedure used to design a dual-resonance metasurface for SHG enhancement is to plot the dependence of the resonance frequency of the quasi-BICs at the FF and SH on the asymmetry parameter *s*. To bring the two resonances within the same frequency range we doubled the frequency of the FF. As a result, a double-resonance phenomenon occurs when the two dispersion curves cross each other. As shown in Figure 5a, for our nonlinear metasurface, the dispersion curves of the quasi-BICs cross at about *s* = 0.65 (see the blue dashed line in Figure 5a). Note that in these calculations the optical losses have been taken into account. In addition, the linear optical near-field within the frequency domain ranging from 685 THz to 725 THz is mainly dominated by a single eigenmode of the optical nanostructure, the field distribution varying only weakly with the frequency. This is so despite the fact that within this frequency range there are about 8 eigenmodes (for a detailed discussion of the mode content of the scattered field at the FF and SH see Section 3 of the Supplementary Material). However, it should be emphasized that the eigenmode that governs the linear scattered field at the SH, corresponding to a TE-polarized incident plane-wave, is not the same as the one that dominates the nonlinear optical field generated at the SH. This is explained by the fact that the linear field scattered at the SH under the excitation with a plane-wave with SH frequency and the nonlinear field generated at SH under the excitation with a plane-wave with FF are produced by different source distributions.

To further study the double-resonant feature of the nonlinear metasurface, we have repeated the transmission analysis for the ideal case in which optical losses in silicon are neglected. As shown in Figure 5b, which depicts the dispersion map of the transmission corresponding to this situation, there are a series of resonances with much larger frequency dispersion and narrower linewidths. As the asymmetry parameter *s* varies from 0 to 1, the quasi-BIC resonance at the FF, indicated by a blue dashed line, crosses the dispersion curves of several resonances around the SH. Nevertheless, the finite linewidth of the resonances makes it difficult to precisely determine the location of the crossing point(s).

To circumvent this problem, we investigated the dependence of the TE-like eigenmodes located nearby the SH (700 THz) on the asymmetry parameter *s* and the results are given in Figure 5c. In the calculation of the eigenmodes, optical losses of silicon are considered so that the conclusions are valid in practical situations. Because of this, the frequency dispersion of resonances in the transmission map in Figure 5b is slightly different from that of the real part of eigenfrequencies in Figure 5c. Moreover, as we have explained, in Section 3 of the Supplementary Material we presented the approach used to identify the eigenmode that governs the nonlinear optical field at the SH. It was found that the eigenmode with frequency of 696.4 THz at *s* = 0 (marked as a purple dashed line in Figure 5c) dominates the nonlinear optical field at the SH, and its frequency band crosses at about *s* = 0.6 the band corresponding to the doubled frequency of the quasi-BIC at the FF. Furthermore, the linewidth of the quasi-BIC at the SH, given by the imaginary part of its eigenfrequency, is indicated by error bars. It shows that this band is relatively broad, which ensures that there is effective coupling with the generated nonlinear polarization in a wide range of values of the asymmetry parameter. Consequently, this analysis supports our conclusion that the designed nonlinear metasurface supports a dual resonance, namely a pair of quasi-BIC modes at the FF and SH. For the sake of completeness, it is noteworthy to mention that the symmetry properties of the interacting optical modes regarding reflection about the plane *x* = 0 allow optical coupling between the nonlinear polarization and other TE-like eigenmodes located around the SH, but these other modes are only weakly excited.

Before we consider the nonlinear optical response of the nonlinear metasurface, we want to discuss the topological nature of the quasi-BICs at the FF and SH (more details on this topic can be found in Section 4 of the Supplementary Material). Thus, our calculations showed that the topological charge, *q*, of the BIC at the FF, determined for *s* = 0, is *q* = 1, which means that this optical mode is topologically nontrivial. The topological characteristics of the quasi-BIC at the SH are more subtle as its frequency lies above the diffraction limit. A simple Fourier analysis shows that this mode contains a zeroth-order and four first-order diffraction channels (more details can be found in the Supplementary Material). Moreover, for *s* = 0, the zeroth-order diffraction channel has topological charge *q* = −1, whereas all first-order diffraction channels are topologically trivial (*q* = 0). Therefore, the optical mode at the SH can be viewed as a quasi-BIC mode, as the zeroth order is completely decoupled from the radiation continuum, whereas all first-order diffraction channels can radiate into the continuum.

### 2.3 Nonlinear optical response of the silicon/MoS_2_ metasurface

Symmetry-breaking dielectric metasurfaces that possess BICs provide a new way for engineering optical resonances with large *Q*-factor, which increases the efficiency of light–matter interaction. Of particular importance in this respect is the possibility to achieve large local field enhancement, which makes dielectric metasurfaces a promising platform to enhance nonlinear optical processes. As a material with large second-order nonlinear susceptibility, single layer MoS_2_ has recently attracted significant research interest. However, with a thickness of a single atomic layer, the nonlinear conversion efficiency it can provide is rather limited. Therefore, given potential applications in sensing, nanophotonics, and nonlinear optics, it is of particular interest to explore to what extent BIC-inspired metasurfaces can enhance nonlinear frequency generation in monolayer-thin MoS_2_.

In this section, we quantify the enhancement of SHG in monolayer MoS_2_ array deposited on top of cruciform silicon metasurface induced by the excitation of the quasi-BIC mode at the FF in the case when a quasi-BIC exists at the SH, too. Due to the centrosymmetric nature of the crystal lattice of silicon, SH is generated from the surface of silicon elements and the monolayer MoS_2_. The enhancement of the contribution to the SH from the former source has been extensively investigated [40]–[44] and as such it is not considered in this study. Therefore, here we include in our analysis only the SHG in the monolayer MoS_2_ array. To this end, to quantitatively characterize the enhancement of the second-order nonlinear optical interaction in monolayer MoS_2_, we use as a reference the SHG intensity corresponding to a cruciform MoS_2_ array suspended in air, whose unit cell is identical to the one deposited on our nonlinear metasurface.

**Figure 6:**
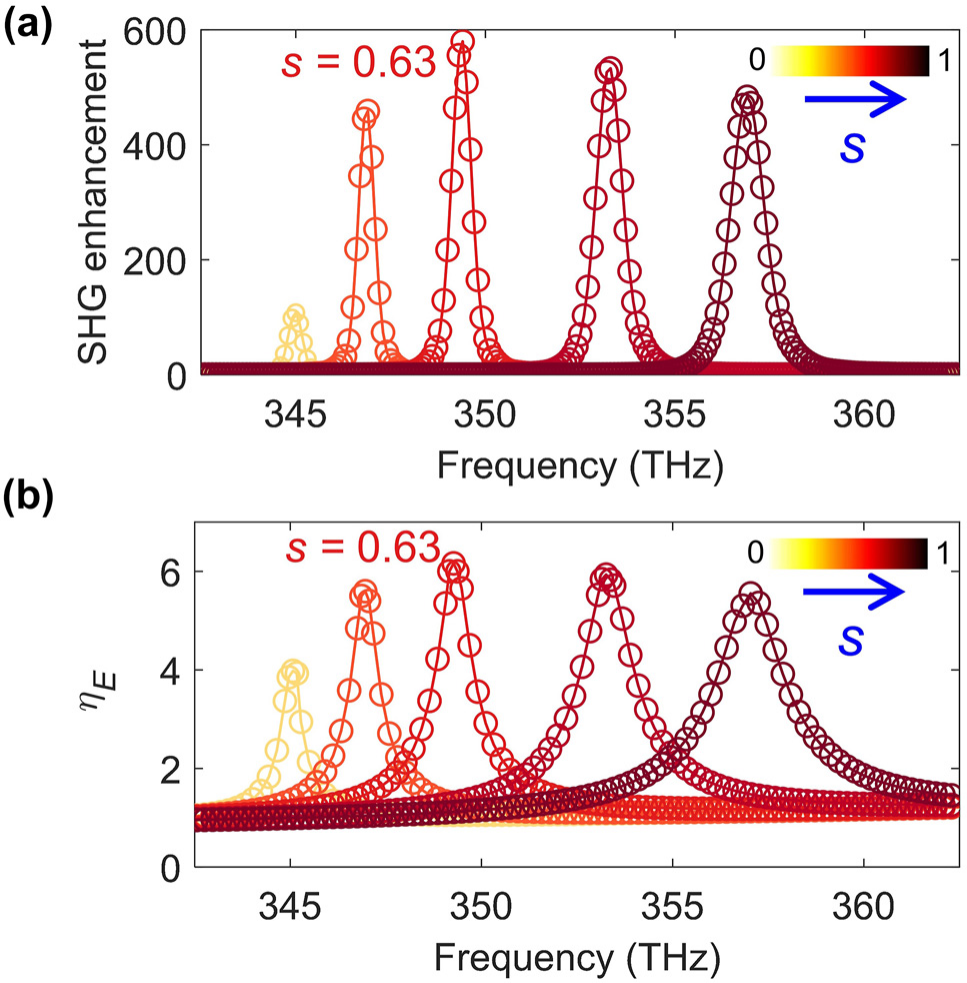
Enhancement of SHG and averaged linear electric field. (a) Spectra of SHG enhancement factor calculated for different values of the asymmetry parameter, with maximum enhancement observed for *s* = 0.63. The reference SHG intensity corresponds to a cruciform MoS_2_ array suspended in air and identical to the one deposited on the nonlinear metasurface. (b) Spectra of the enhancement factor *η*_*E*_ of the averaged electric field amplitude within the monolayer MoS_2_ under TE-polarized plane-wave illumination, calculated for different values of the asymmetry parameter.

The frequency dependence of the enhancement of the SHG, determined for several values of the asymmetry parameter *s*, is presented in Figure 6a. As it can be seen in this figure, the maximum SHG enhancement in monolayer MoS_2_ is as large as 600 and is reached for *s* = 0.63 and frequency of 699 THz. This frequency is exactly twice as large as the frequency of the quasi-BIC mode at the FF, as per Figure 3a. The narrow spectral resonance displayed by the SHG enhancement factor indicates a strong nonlinear light–matter interaction. Importantly, the enhancement of SHG vanishes for *s* = 0, due to the complete decoupling between the BIC of the symmetric metasurface and incident electromagnetic waves. As a further observation, note that the frequency of the maximum SHG enhancement in Figure 6a is nearly identical to that at which the second-order susceptibility of monolayer MoS_2_ reaches its maximum (see Section 2 of the Supplementary Material).

Given the relation between the nonlinear polarization **P**_*nl*_ and the linear optical field, it is expected that the enhanced light–matter interaction at the FF results in increased SHG. To illustrate this idea, we calculated for several values of the asymmetry parameter the averaged enhancement factor of the linear optical field within the MoS_2_ monolayer defined by

(1) $$\eta_{E}=\frac{1}{A_{0}}\underset{S}{\int }\frac{\left|E\left(r,\omega \right)\right|}{E_{0}}\;\text{d}r,$$

where *E*_0_ is the amplitude of the impinging field, *A*_0_ is the area of the monolayer MoS_2_, and *S* is the middle plane of the cruciform-shaped MoS_2_ monolayer. The results of these calculations, plotted in Figure 6b, demonstrate the fact that the averaged local field enhancement, *η*_*E*_, and the SHG intensity reach their maximum for the same value of the asymmetry parameter, *s* = 0.63. In addition, when compared to the asymmetry-dependent band dispersion curves in Figure 5c, the asymmetry parameter *s* corresponding to the largest SHG enhancement is almost identical to that at which the double-resonance condition is satisfied, the difference being about 3 %. This finding suggests that the double-resonance phenomenon plays a key role in the enhancement of the SHG.

Previous investigations [35], [37] have shown that the maximum enhancement of the local optical field upon excitation of quasi-BICs and, consequently, the largest SHG enhancement, is achieved in the critical coupling regime whereby the radiative and nonradiative losses become equal. The balance of the optical losses in the two loss channels can be achieved by adjusting the asymmetry of the metasurface. In this work, we use a versatile semi-analytic theoretical model [42] based on the eigenmode expansion method to reveal the main characteristics of the dependence of the SHG enhancement on the asymmetry parameter. The model allows the quantitative analysis of each stage in the frequency up-conversion process, thus conveying the underlying physics in an explicit way. Within the framework of this model, at the FF (SH) there is only one eigenmode with complex frequency *ω*_1_ − *iγ*_1_ (*ω*_2_ − *iγ*_2_) and field distribution **E**_1_(**r**) (**E**_2_(**r**)). In our case, the two optical modes are represented by the two quasi-BICs at the FF and SH. Under these circumstances, the optical power at the SH, *P*_SH_(Ω), is given by [42] :

(2) $$P_{\text{SH}}\left(2\omega \right)={\left[P\left(\omega \right)\kappa_{1}\left(\omega \right)Q_{1}L_{1}\left(\omega \right)\right]}^{2}\kappa_{12}Q_{2}L_{2}\left(2\omega \right)\kappa_{2}\alpha \left(2\omega \right).$$

Here, *P*(*ω*) is total incident power at the FF, *κ*_1_(*ω*) is the coupling coefficient between the incoming light and the quasi-BIC mode **E**_1_(**r**), *κ*_12_ is the optical coupling coefficient between the modes **E**_1_(**r**) and **E**_2_(**r**), *κ*_2_ is the out-coupling coefficient at the SH, *α*(2*ω*) is a smoothing factor, and *Q*_1,2_ and *L*_1,2_ are the *Q*-factors and spectral mismatch functions of the two quasi-BICs, respectively. An exact definition and discussion of the physical parameters in Eq. (2) are presented in Section 5 of the Supplementary Material.

**Figure 7:**
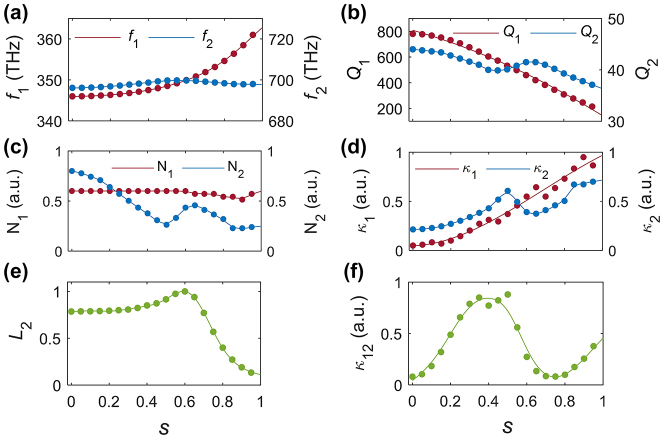
Influence of asymmetry parameter *s* on the factors that determine *P*_SH_(2*ω*). (a) Frequency of the quasi-BICs versus *s*. (b) Quality-factors of the quasi-BICs versus *s*. (c) Normalization constants of the quasi-BICs versus *s*. (d) Coupling coefficients *κ*_1,2_ versus *s*. (e) Dependence on *s* of the spectral overlap function *L*_2_. (f) Dependence of the nonlinear coupling coefficient *κ*_12_ on *s*. In all these plots, the label “1” (“2”) refers to the quasi-BIC at the FF (SH).

The theoretical model summarized by Eq. (2) has been used to calculate the optical power generated at the SH by the monolayer MoS_2_ cruciform array placed on top of the silicon metasurface. More specifically, we have determined the dependence on the asymmetry parameter of the various factors entering in Eq. (2). The results of these calculations are summarized in Figure 7. Thus, Figure 7a and b shows that the quasi-BIC at the FF is much more dispersive and has a larger *Q*-factor than the one at the SH. This latter finding is explained by the fact that optical losses in silicon are much larger at SH as compared to losses at FF. Moreover, as expected, the *Q*-factor of the quasi-BICs decreases when the asymmetry of the metasurface increases, with a much steeper decrease being observed in the case of the FF. This behavior underlines the difference in the underlying physics that characterizes the two resonances: whereas the *Q*-factor of the quasi-BIC at the FF is intimately related to the symmetry properties of the metasurface, the *Q*-factor of the quasi-BIC at the SH is primarily determined by the optical losses in the silicon components. In addition, the value of the *Q*-factor of the quasi-BIC at the FF extracted from the Fano formula is in good agreement with the *Q*-factor calculated from eigenmode analysis (compare Figures 3b–7b).

In the theoretical framework in which Eq. (2) is derived one introduces two frequency-dependent constants, *N*_1,2_, which are used to normalize the eigenmodes (for details, see Section 5 of the Supplementary Material). The dependence of these constants on the asymmetry parameter is shown in Figure 7c. Moreover, in Figure 7d we show the dependence on the asymmetry of the metasurface of the coupling coefficient, *κ*_1_ (*κ*_2_), between the free-space radiation modes and the quasi-BIC mode at the FF (SH). Interestingly enough, it can be seen that the coupling coefficient *κ*_1_ vanishes at *s* = 0, which proves the fact that in the case of a symmetric metasurface the BIC is completely decoupled from the free-space radiation modes.

In Figure 7e, we plot the dependence on the asymmetry parameter of the spectral line shape, *L*_2_(2*ω*), evaluated at a frequency twice as large as the frequency of the quasi-BIC at the FF. This figure shows that *L*_2_ is maximum at about *s* = 0.6, where, as per Figure 7a, the double-resonance phenomenon occurs. Moreover, given its definition (see Section 5 in the Supplementary Material), the spectral line shape factor *L*_1_ ≡ 1. Finally, in Figure 7f we plot the dependence on *s* of the nonlinear optical coupling coefficient, *κ*_12_, between the quasi-BIC modes at the FF and SH; it reaches a maximum value at *s* ≈ 0.5.

**Figure 8:**
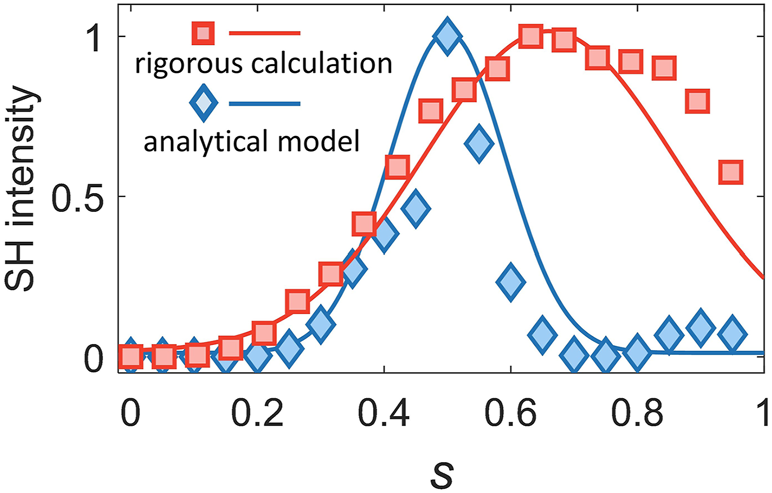
Comparison between normalized maximum SHG intensities determined *via* rigorous full-wave numerical simulations and the analytic model based on Eq. (2). The two solid lines were obtained by fitting the data with a Gaussian function.

We have collected all the results presented in Figure 7 and, using Eq. (2), have calculated the SHG from the monolayer MoS_2_ cruciform array deposited onto our silicon metasurface. The results of this analysis are presented in Figure 8, together with the SHG dependence on the symmetry parameter computed *via* rigorous full-wave simulations of the nonlinear optical response of the silicon/MoS_2_ composite metasurface. This figure reveals a relatively good agreement between the predictions of the two approaches, with the maximum SH intensity of the analytic and numerical results being observed at *s* = 0.5 and *s* = 0.63, respectively. This difference can be traced to the assumption that the interacting fields at the FF and SH are single modes, meaning that the radiative modes forming the spectral background are neglected. Additionally, the calculated results of *κ*_2_ (*κ*_12_) demonstrate a local maximum (maximum) value at *s* = 0.5, and the linewidth of the SH mode in Figure 5c implies a weak coupling between the FF and SH modes as *s* ⪆ 0.75. As a result, these observations explain to some extent the discrepancy between the analytical and numerical results. Nevertheless, compared to the rigorous but extremely time-consuming full-wave numerical simulations, the analytical model provides a more efficient approach to investigate the SHG from nonlinear metasurfaces and a convenient and intuitive way to identify and separate the relevant physics that plays the main role in the generation of the SH.

### 2.4 Nonlinear homogenization for monolayer MoS_2_

Nonlinear susceptibility is one of the most commonly used physical quantities to characterize the nonlinear optical properties of an optical structure. In this section, the effective second-order susceptibility $\chi_{e\mathrm{ff}}^{\left(2\right)}$ of our nonlinear metasurface is evaluated using a versatile homogenization method we have recently developed [45], [46] (see details of this method in Section 6 of the Supplementary Material). Using this effective second-order susceptibility, we subsequently computed the spectrum of the enhancement factor of SHG interaction, defined as $\eta \left(\omega \right)=|\chi_{e\mathrm{ff}}^{\left(2\right)}\left(\omega \right)/\chi_{{MoS}_{2}}^{\left(2\right)}\left(\omega \right)|$ .

Due to the *D*_3*h*_ point-group symmetry of MoS_2_, its second-order nonlinear susceptibility tensor has only one independent and nonzero component [14]: $\chi_{{MoS}_{2}}^{\left(2\right)}\equiv \chi_{xxx}^{\left(2\right)}=-\chi_{xyy}^{\left(2\right)}=-\chi_{yyx}^{\left(2\right)}=-\chi_{yxy}^{\left(2\right)}$ . Importantly from a theoretical point of view, because the quasi-BIC mode at the FF is antisymmetric with respect to reflection symmetry about the *x* = 0 plane, the only component of the monolayer MoS_2_ nonlinear susceptibility tensor that needs to be considered is $\chi_{xxx}^{\left(2\right)}$ .

**Figure 9:**
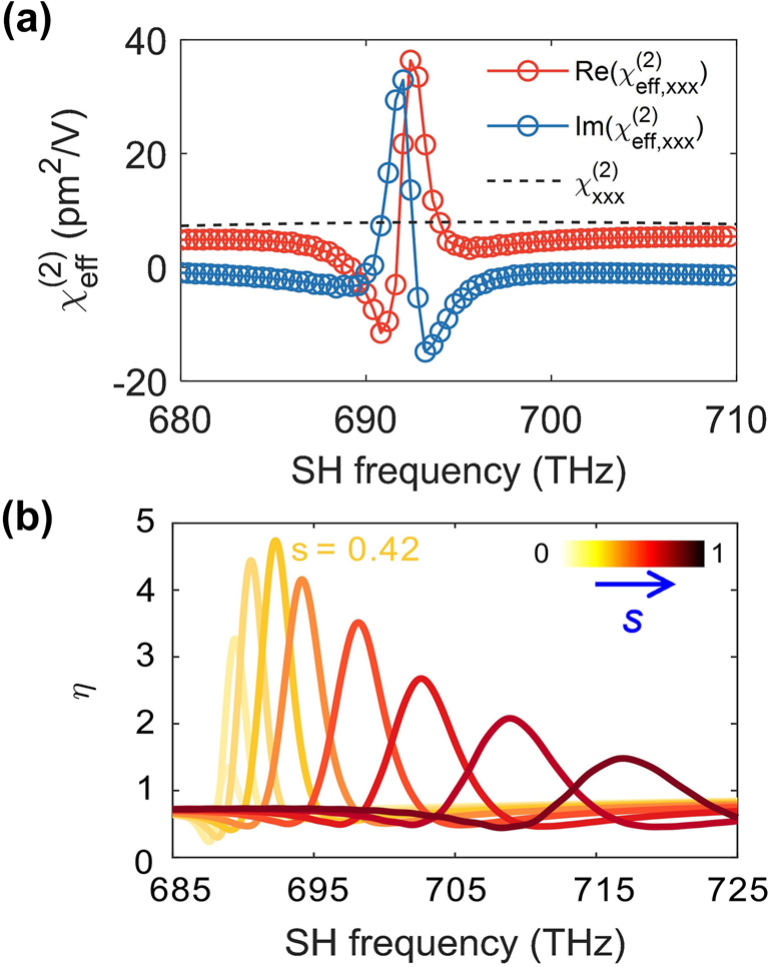
Homogenization of second-order susceptibility. (a) Intrinsic and effective second-order susceptibility of MoS_2_ determined for *s* = 0.42. (b) Enhancement factor *η* of second-order susceptibility of monolayer MoS_2_ versus SH frequency, computed for different values of *s*. A maximum *η* is achieved for *s* = 0.42.

The results of this homogenization procedure are depicted in Figure 9. Thus, in Figure 9a, we show the spectra of the real and imaginary parts of the nonlinear effective susceptibility, $\chi_{e\mathrm{ff}}^{\left(2\right)}$ , of the monolayer MoS_2_ cruciform array, determined for the asymmetry parameter *s* = 0.42. For comparison, we also plot in this figure with a dashed black line the frequency dependence of intrinsic second-order nonlinear susceptibility of monolayer MoS_2_, $\chi_{xxx}^{\left(2\right)}$ . These plots clearly demonstrate the resonant nature of $\chi_{e\mathrm{ff}}^{\left(2\right)}$ , which is due to the resonant enhancement of the local field induced by the quasi-BIC at the FF. In particular, the resonance frequency of $\chi_{e\mathrm{ff}}^{\left(2\right)}$ is twice as large as the frequency of the quasi-BIC mode corresponding to *s* = 0.42 (see Figure 3a). This indicates that the large enhancement of $\chi_{e\mathrm{ff}}^{\left(2\right)}$ of the MoS_2_ cruciform array is due to the light–matter interaction boosted by the quasi-BIC at the FF.

As demonstrated in Figure 9b, the enhancement factor *η*(*ω*) shows strong dependence on the asymmetry parameter *s*, chiefly due to the large frequency dispersion of the quasi-BIC mode. The enhancement factor reaches a maximum value *η* ≈ 5 when *s* = 0.42 and Ω = 692.4 THz. Comparing the plots in Figures 6a and 9b, one can see that both the SHG intensity and second-order susceptibility enhancement spectra display resonances whose linewidth gradually increases as *s* increases. This trend is the result of the fact that the *Q*-factor of the quasi-BIC mode at the FF decreases when *s* increases. Interestingly enough, the asymmetry parameter at which the largest SHG enhancement is achieved (*s* = 0.63, see Figure 6a) is different from the one that corresponds to the maximum enhancement of the second-order susceptibility (*s* = 0.42, see Figure 9b). This difference is explained mainly by the fact that the SHG enhancement is determined by both the light coupling to the modes at the FF and SH and the local field enhancement at these frequencies, whereas the enhancement of the effective second-order susceptibility is due only to the latter effect.

## 3 Conclusions

In conclusion, we have demonstrated giant enhancement of SHG from monolayer MoS_2_ deposited on an asymmetric silicon metasurface, originating from the strong light–matter interaction boosted by the excitation of quasi-bound states in the continuum at both the fundamental frequency and second-harmonic. In particular, we show that the *Q*-factor of the two resonances and local field enhancement can be engineered by carefully tuning the asymmetry of the metasurface. Moreover, we find that a double-resonance condition can be fulfilled for a certain value of the asymmetry parameter, resulting in strong enhancement of the intensity of the generated second-harmonic. Thus, our analysis shows that the second-harmonic generated in a monolayer MoS_2_ deposited onto the silicon metasurface is up to three orders of magnitude larger than that of the same MoS_2_ cruciform array but suspended in air. In addition, to gain deeper physical insights into the reasons behind the giant enhancement of the intensity of the generated second-harmonic, we also employed a semi-analytic eigenmode expansion method and observed a good agreement between predictions of this semi-analytic model and rigorous numerical simulations. Moreover, a powerful homogenization method is used to extract the effective second-order nonlinear susceptibility of the proposed nonlinear metasurface. It was found that the spectral resonances of the effective nonlinear susceptibility of the metasurface coincide with the frequency of symmetry-protected bound-states in the continuum of the optical structure.

Our designed nonlinear optical metasurface can spur and guide further research in nonlinear optical systems and new photonic applications. In particular, the ideas developed in this work could have great impact to ultra-thin optical devices for nonlinear holography applications [47], [48], by facilitating efficient optical beam generation and holographic image reconstruction at optical frequencies other than the excitation fundamental frequency. More specifically, the enhanced nonlinear optical interactions facilitated by the excitation of optical resonances at both the fundamental frequency and second harmonic would greatly reduce the optical power at which these active photonic devices operate.

## Supplementary Material

Supplementary Material Details
