# Supplementary material for: Giant second-harmonic generation in monolayer MoS2 boosted by dual bound states in the continuum
Source: Nanophotonics. 2024 Jul 11;13(18):3437–48. doi: 10.1515/nanoph-2024-0273 (PMC11501448; doi:10.1515/nanoph-2024-0273)
Supplement: Supplementary file 1 — Supplementary Material Details [file j_nanoph-2024-0273_suppl_001.pdf]

Research Article

Ji Tong Wang, Jian Wei You, and Nicolae C. Panoiu*

Supplementary Material: Giant second-harmonic generation in monolayer MoS_2 boosted by dual bound states in the continuum

1 Numerical methods

In this section we discuss the numerical methods used to model the linear and nonlinear optical properties of the optical structures considered in this paper. For linear simulations, to calculate the transmission map with respect to the angle of incidence and wavelength of the plane-wave excitation, we used the rigorous coupled-wave analysis (RCWA) implemented in DiffractMOD [1], a commercial software produced by Synopsys. Using the calculated transmission spectra, the Q-factor can be determined from the Fano formula (as described in the Main Text) *via* the Levenberg-Marquardt algorithm [2, 3] based on the nonlinear least squares method. In addition, the eigenmode analysis of the designed nonlinear metasurface was performed using the eigenmode solver of COMSOL Multiphysics [4]. The computed results, namely the resonant features of the optical system, obtained from RCWA agree well with the predictions of COMSOL Multiphysics, with less than 1 % difference of the values of resonance frequency.

For nonlinear simulations, we employ the time-domain solver in CST Microwave Studio [5]. In particular, we used this software to investigate the optical field distribution in the proposed nonlinear metasurface. To be more specific, a nonlinear simulation takes three steps: ***Step-1***, we calculate the near-field distribution at the FF throughout the monolayer MoS_2 within the frequency range of interest; ***Step-2***, we evaluate the nonlinear surface currents using the FF near-field distribution calculated in *Step-1* and the second-order nonlinear optical susceptibility of MoS_2; and ***Step-3***, the nonlinear surface currents obtained in *Step-2* are employed as nonlinear excitation source in the nonlinear simulation to excite the metasurface and generate the SH field distribution [6].

All simulations are carried out in one unit cell with Bloch (periodic) boundary conditions along the x- and y-axes. In the case of simulations in COMSOL Multiphysics and CST Microwave Studio, perfect matching layers (PMLs) are used along the z-axis, so as to avoid nonphysical back reflections.

Ji Tong Wang, University College London, London, United Kingdom, jitong.wang@ucl.ac.uk; https://orcid.org/0009-0005-5693-1749
Jian Wei You, Southeast University, Nanjing, China, jvyou@seu.edu.cn; https://orcid.org/0000-0001-5761-9507
***Corresponding author: Nicolae C. Panoiu,** University College London, London, United Kingdom, n.panoiu@ucl.ac.uk; https://orcid.org/0000-0001-5666-2116

2 Material properties

In this section, the optical properties of the materials of which the nonlinear metasurface is made are described. Thus, the frequency dispersion of the complex permittivity of silicon [7] within a frequency range of interest is plotted in Figure 1. It shows that the optical loss of silicon in the range of FF, described by the imaginary part of the permittivity, is relatively large. Therefore, it is expected that the Q-factor of BIC modes evaluated in the case when optical losses are taken into account has a relatively small value. In the lossless case, however, the Q-factor of BICs becomes infinite, its linewidth becoming vanishingly small. Additionally, it also demonstrates that the optical loss of silicon is considerable within the range of the SH (around 700 THz), leading to significant losses of SHG.

For the quartz substrate, we neglect its frequency dispersion around both FF and SH, and assume that its index of refraction is $1.4602 + i0.0012$ and $1.473 + i0.0025$, respectively [8]. Moreover, the complex permittivity $\epsilon(E)$ of the monolayer MoS_2 is assumed to be a superposition of N Lorentzian functions. Expressed as a function of the photon energy, $E = \hbar\omega$, we write it as: [9]

$$\epsilon(E) = 1 + \sum_{k=1}^{N} \frac{f_k}{E_k^2 - E^2 - iE\gamma_k}. \quad (1)$$

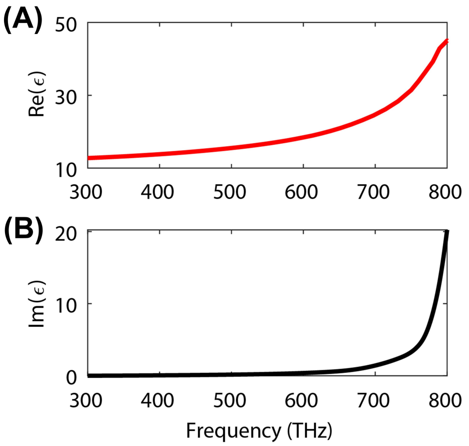

Figure 1: Real and imaginary part of the permittivity of silicon.

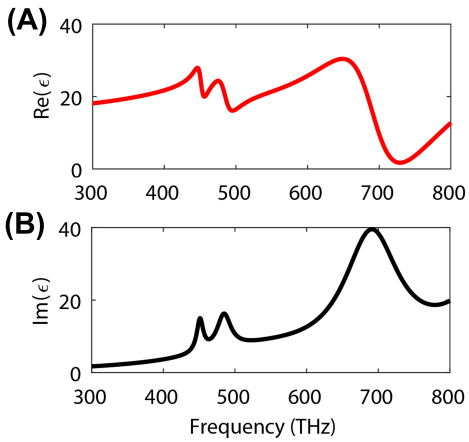

Figure 2: Real and imaginary parts of the permittivity of monolayer MoS_2.

Here, f_k, γ_k and E_k are the oscillator strength, linewidth, and spectral resonance energy of the kth oscillator, respectively. The model parameters [10] are extracted by fitting experimental data to Eq. (1) and the corresponding complex permittivity of monolayer MoS_2 is presented in Figure 2.

Due to the centrosymmetric nature of the lattice of the silicon crystal, SHG in bulk silicon is generally forbidden. By contrast, due to its symmetry belonging to the D_{3h} point-group, the monolayer MoS_2 has a nonzero second-order nonlinear susceptibility, whose third-rank tensor has only one independent, nonvanishing element [11]:

$$\chi^{(2)}_{MoS_2} \equiv \chi^{(2)}_{xxx} = -\chi^{(2)}_{xyy} = -\chi^{(2)}_{yyx} = -\chi^{(2)}_{yxy}, \quad (2)$$

where x- and y-axes correspond to armchair and zigzag directions, respectively.

In Figure 3, the second-order sheet nonlinear susceptibility of monolayer MoS_2 is presented in a frequency range close to that considered in our paper. It is worth noting that the nonlinear susceptibility of $\chi^{(2)}_{MoS_2}$ has a maximum at about 700 THz, which is almost equal to the wavelength at which maximum SHG is observed in our nonlinear metasurface.

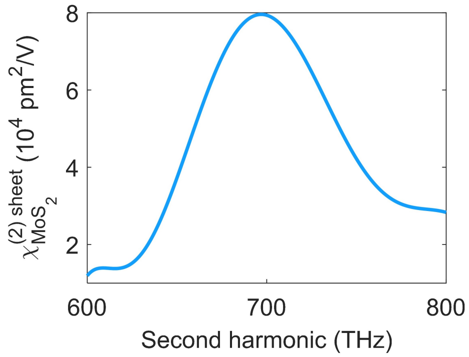

Figure 3: Second-order sheet susceptibility of monolayer MoS_2.

3 Modal content of the scattered fields

The use of the eigenmode expansion method for the semi-analytical calculation of SHG relies on the correct identification of the dominant optical modes in the linear and nonlinear scatter fields. Therefore, in this section, we analyze the eigenmodes present in the scattered fields at the FF and SH and compare them with the optical modes supported by the optical metasurface in the corresponding frequency domains.

For the dominant mode at the FF under TE-polarized plane-wave illumination, it is clear that in the case of the metasurface with symmetric meta-atoms the BIC observed at 345.8 THz, at the Γ-point, is the dominant mode in the scattered field at the FF. In particular, as can be seen in Figure 3A in the Main Text, there are no other modes nearby this optical resonance. However, at the SH, one expects that there are several modes as the spectral density of modes increases when the frequency increases. In addition, the relatively large linewidth of these eigenmodes originating from the increased optical loss of silicon at SH can induce a spectral overlap of these modes. Therefore, to precisely find out the eigenmode that governs the nonlinear scattered field generated by nonlinear surface currents at the surface of atomically thin MoS_2 layer, we present the frequency and electric-field distributions, $|E_x|$ and $|E_y|$, of a few TE-like eigenmodes

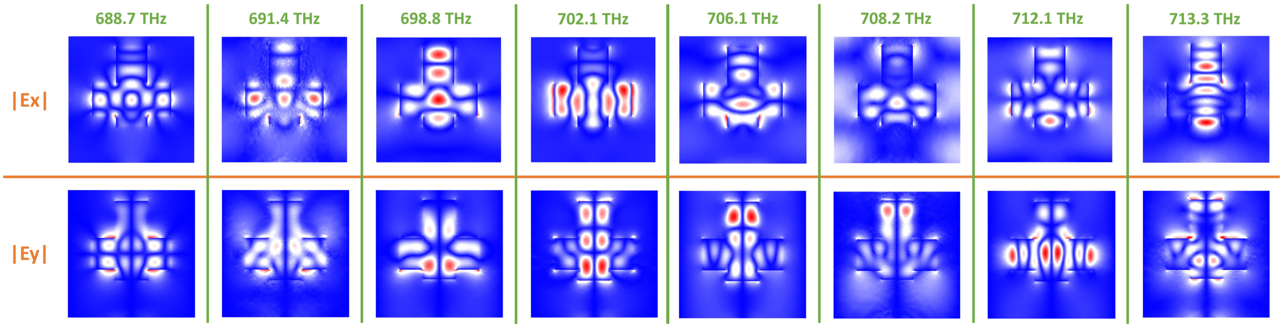

Figure 4: Spatial distribution of the in-plane components of the electric fields determined in the (x, y)-plane for the eigenmodes in the vicinity of the SH at $s = 0.63$. For each mode, the real part of the eigenfrequency is given at the top of the field patterns.

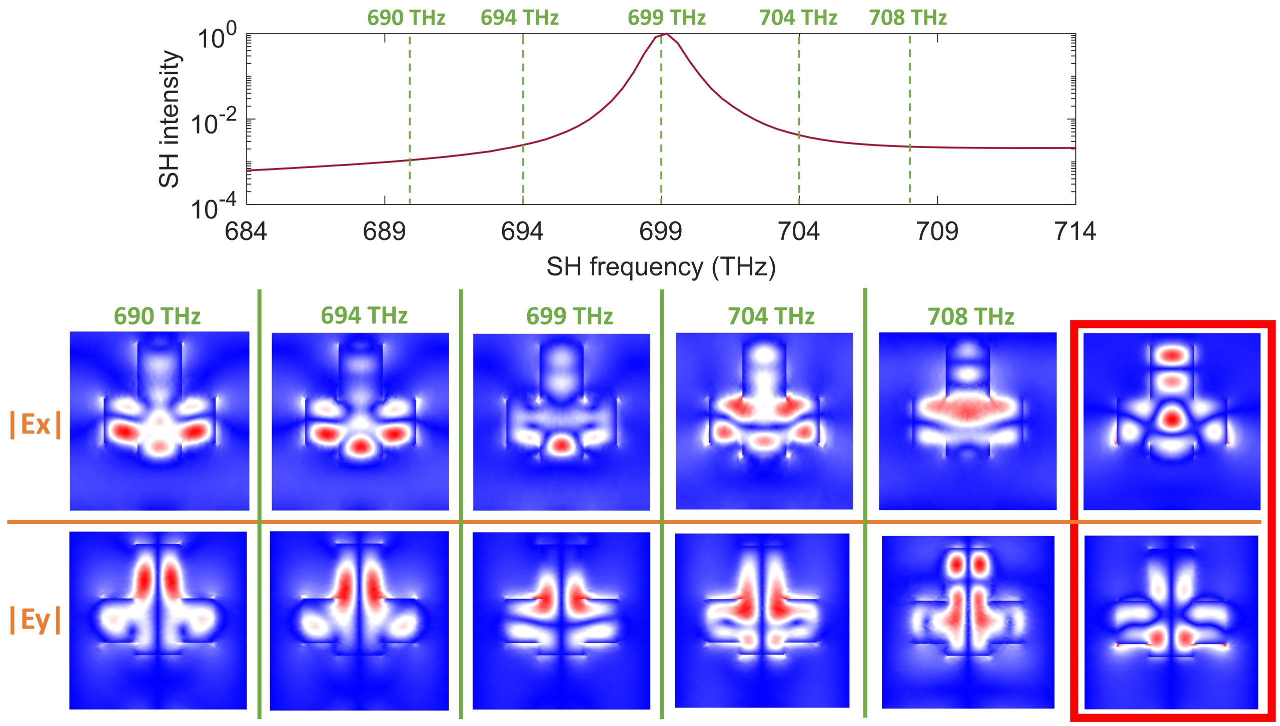

Figure 5: Dispersion of the generated SH intensity from the designed nonlinear optical metasurface at $s = 0.63$. The in-plane components of the nonlinear near-field in the (x, y)-plane are presented at five frequency points, denoted by green dashed lines in the top panel, around the resonance peak of SH intensity. The field distributions inside the red frame correspond to the eigenmode with frequency of $698.8\,\mathrm{THz}$ in Figure 4.

lying in the range of the SH determined in the (x, y)-plane across the middle section of silicon components, as per Figure 4. This eigenmode analysis is carried out for the geometric asymmetry parameter $s = 0.63$, at which maximum SH emission is observed. Here, only TE-like modes are discussed because of their symmetry compatibility with the nonlinear polarization when the mirror symmetry about the plane $x = 0$ is taken into consideration.

Next, to determine the main mode present in the nonlinear scattered field, one compares the field profiles of the eigenmodes and computed nonlinear scattered field. To this end, for $s = 0.63$, we calculated the spectral dependence of the normalized field intensity at the SH (Figure 5). Due to the presence of the quasi-BIC mode at the FF, the SH intensity presents a resonant feature with central frequency at twice the frequency of the FF mode. Then, to obtain a more complete picture of the nature of the nonlinear scattered field, we chose five frequency points ($690\,\mathrm{THz}$, $694\,\mathrm{THz}$, $699\,\mathrm{THz}$, $704\,\mathrm{THz}$, and $708\,\mathrm{THz}$) around this resonance frequency and plotted the near-field distri-

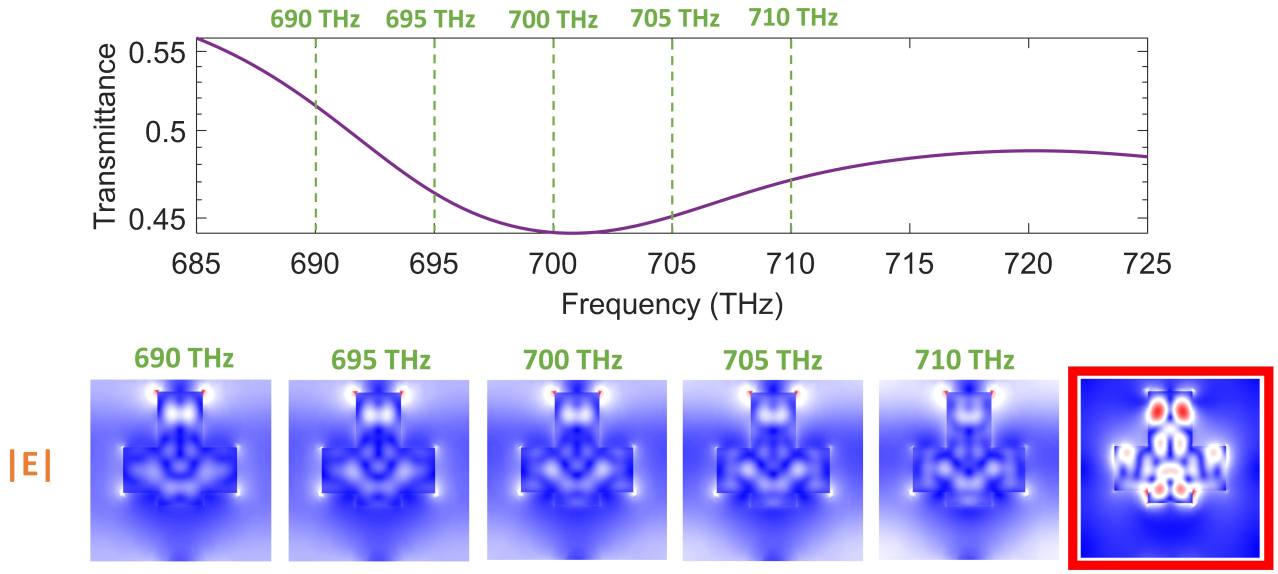

Figure 6: Linear optical transmittance of the silicon metasurface under TE-polarized plane-wave excitation at $s = 0.63$. The amplitude of the electric field on the x–y plane of the unit cell is shown at five frequency points, marked in the top panel by green dashed lines. For comparison, the distribution of the optical field of the eigenmode at $698.8\,\mathrm{THz}$ is shown inside the red frame.

butions, $|E_x|$ and $|E_y|$, in the (x, y)-plane across the middle section of silicon components. From these field patterns, it can be seen that the nonlinear optical fields at different frequencies have roughly similar patterns, which suggests that there exists a dominant eigenmode at the SH. Moreover, by comparing the nonlinear optical field with the modes around the SH shown in Figure 4, one can conclude that the eigenmode with the frequency of 698.8 THz is the dominant optical mode in the nonlinear scattered field. For a better comparison, we also present the field distribution of this mode, shown in Figure 5 surrounded by a red frame. The dispersion of this mode with respect to s is given in Figure 5C of the Main Text, from which the crossing of FF and SH bands can be observed at $s = 0.59$. Importantly, the large value of the linewidth of the SH mode leads to mode overlap over a wide range of s.

For the sake of completeness, we also discuss the method commonly used in doubly resonant nanostructures for SHG, namely the observation of double-resonance phenomena from optical transmission spectra. In our work, the dispersion map of transmission with optical losses of silicon included is shown in Figure 5A of the Main Text. In this figure, there exists a resonance valley that crosses the quasi-BIC mode at the FF at $s \approx 0.65$. To identify the mode that dominates the linear scattered field under TE-like plane-wave excitation, we present in Figure 6 the transmittance in the case of $s = 0.63$ (maximum SHG is achieved for this value of the asymmetry parameter) and the amplitude of the electric field calculated for several values of the FF frequency. As can be seen in this figure, the near-field hardly changes as the frequency varies from 690 THz to 710 THz. Compared to the spatial profiles of the optical field of the eigenmodes within this frequency range of interest, the mode with an eigenfrequency of 706.1 THz (the electric field profile of the mode is shown in a red frame in Figure 6) exhibits a similar field pattern, indicating that it is the dominant mode in the linear scattered field.

4 Topological nature of the interacting optical resonances

In this section we discuss the topological properties of the interacting modes at the FF and SH. To characterize the reduced energy leakage arising from symmetry protection, only the radiative Q-factor is studied and therefore the optical losses in the silicon components of the metasurface are not included in the calculation of the Q-factor. This is a reasonable approximation as the topological properties of BICs are independent on the intrinsic material optical losses.

To start with, the topological charge, q, of the resonant mode (BIC) at the FF, corresponding to the case

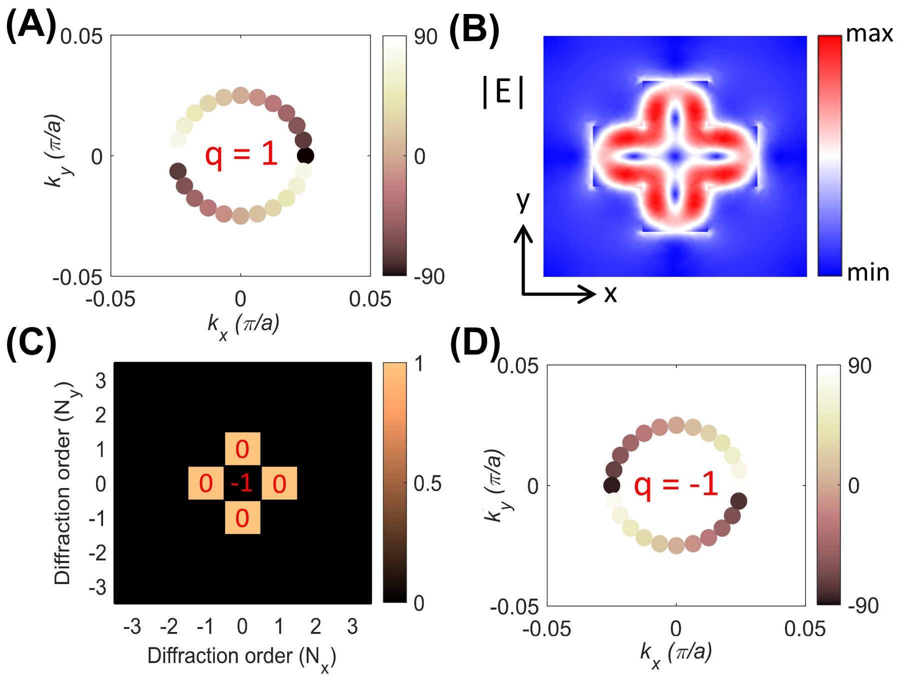

Figure 7: Topological analysis of interacting optical resonances and mode profile of the SH mode. (a) Evolution of the far-field polarization angle along a **k**-circle centered at the BIC at the FF. Topological charge $q = 1$. (b) The amplitude of the electric field of the SH mode inside the unit cell, determined in the (x, y)-plane. (c) Far-field Fourier analysis of the SH mode in the air region above the metasurface. Topological charge is $q = -1$ for (0,0) order and $q = 0$ for $(1, 0)$, $(-1, 0)$, $(0, 1)$, and $(0, -1)$ orders. (d) Evolution of the far-field polarization angle of the zeroth-order diffraction channel of the SH mode along a **k**-circle centered at the mode. In all these calculations, $s = 0$ and $\mathfrak{Im}(\epsilon_{\text{Si}}) = 0$.

of $s = 0$, is determined by using the expression [12, 13]

$$q = \frac{1}{2\pi} \oint_C \nabla_{\mathbf{k}} \phi(\mathbf{k}) \cdot d\mathbf{k}. \tag{3}$$

In our calculations, C was chosen to be a circle with radius of $0.025\pi/a$ that encircles a BIC-type mode along the counterclockwise direction and $\phi(\mathbf{k}) = \arg[S_1(\mathbf{k}) + iS_2(\mathbf{k})]$ is the polarization angle with S_1 and S_2 being the Stokes parameters of the far-field polarization vector in the air region above the metasurface, projected on the (s, p)-plane [13]. As can be observed from Figure 7A, these calculations lead to $q = 1$, suggesting the topological nature of the BIC. Moreover, the numerically calculated Q-factor of the BIC is infinitely large if the imaginary part of the permittivity of silicon is set to zero, which further confirms the topological nature of the BIC-type mode.

We next considered the SH mode that governs the scattered nonlinear optical field corresponding to $s = 0$, in the case when the optical losses in silicon were neglected, and computed the topological charge and Q-factor. It was found that the SH mode has a frequency of 697.1 THz and Q-factor of 5288. The corresponding near-field pattern determined in a plane passing through the middle of the silicon meta-atoms is presented in Figure 7B. At the operating frequency of 697.1 THz, there exist 5 diffraction channels in the air region above the metasurface and 9 diffraction channels in the substrate region below the metasurface. Moreover, the Q-factor of this mode is unusually large given that the optical energy can leak into all existing diffractive channels. To understand the reason for this large value of the Q-factor, we performed the Fourier analysis of the far-field in the air domain, and the calculated results are illustrated in Figure 7C. From this figure it can be observed that the energy leakage from the zeroth diffraction order vanishes, thus explaining the large value of the Q-factor. Moreover, the radiation into the 4 first-order diffraction channels remains largely suppressed.

The underlying physics of the vanishing energy leakage into the zeroth diffraction channel of the SH mode is explained by its incompatibility with the symmetry of the propagating waves of the continuum. That is to say, topological properties of the mode can be defined in a similar way as in the case of the BIC at the FF. To confirm this idea, we determined the topological charge, q, of the zeroth diffraction of the

SH mode using Eq. (3). The results, presented in Figure 7D, clearly show that the far-field polarization of the SH mode has a phase change of -2π along the circular path, indicating that $q=-1$. In addition, we analyzed the topological properties of the four first-order diffraction channels, and found out that the computed topological charges are all equal to zero. Therefore, this SH mode can be viewed as a quasi-BIC mode as the zeroth order completely decouples from the continuum, whereas all first-order diffraction channels can radiate into the continuum. If the structural symmetry of the metasurface is broken, namely $s \neq 0$, the zeroth diffraction channel becomes radiative and leaks energy into the far-field. For instance, the Q-factor of this mode drops to 670 at $s = 0.63$, mainly because of the additional outgoing energy emitted from the zeroth-order channel.

In practice, the optical losses of silicon should be taken into consideration and thus the Q-factor of both FF and SH modes has a finite and smaller value as compared to the lossless case (see Figure 7B in the Main Text). Nevertheless, both the BIC at the FF, which governs the linear scattered field, and the quasi-BIC mode at the SH that dominates the scattered nonlinear field have a topological nature.

5 Eigenmode expansion method for computing the second-harmonic generation

In this section, we briefly review an approach for calculation of the SHG intensity using an eigenmode expansion method[14]. The main idea is to expand the Green function, $\hat{\mathbf{G}}_\omega$, of the optical resonator into the direct (dyadic) product of the electric-field eigenfunctions, $\mathbf{E}_j(\mathbf{r})$

$$\hat{\mathbf{G}}_\omega(\mathbf{r},\mathbf{r}') = \sum_j \frac{c^2}{2N_j} \frac{\mathbf{E}_j(\mathbf{r}) \otimes \mathbf{E}_j(\mathbf{r}')}{\omega(\omega-\omega_j+i\gamma_j)}, \tag{4}$$

where $\omega_j - i\gamma_j$ is the complex eigenfrequency and N_j represents a normalization constant for $\mathbf{E}_j(\mathbf{r})$. The expression for this constant is [15]

$$N_j = \int_V \epsilon(\mathbf{r})\mathbf{E}_j \cdot \mathbf{E}_j \, dV + \frac{c^2}{2(\omega_j - i\gamma_j)} \oint_S \left[\mathbf{E}_j \cdot \frac{\partial}{\partial r} r \frac{\partial \mathbf{E}_j}{\partial r} - r \left(\frac{\partial \mathbf{E}_j}{\partial r} \right)^2 \right] dS, \tag{5}$$

where the region of volume integral in Eq. (5) is chosen as an arbitrary volume V which contains all inhomogeneities of $\epsilon(\mathbf{r})$ and the optical modes of the optical system, and the surface integral is taken over the boundary of V, $S = \partial V$. Since the electric-field eigenfunctions decay in space with a ratio of at least $1/r^2$, one can also choose the whole space as the integration region of the normalization constant N_j, whereas in this case the surface integral will vanish at infinity.

In our work, the numerical simulation is performed within one unit cell employing periodic boundaries along x- and y-axis and PMLs along the z-axis. In addition, both the upper free-space region and lower quartz substrate are terminated with PMLs so as to mimic waves propagating to infinite. Therefore, we can choose the integration volume for the normalization constant N_j to be one unit cell and the corresponding surface integral is reduced to integration across two surfaces normal onto the z-axis. Then, Eq. (5) can be converted from spherical to Cartesian coordinates as

$$N_j = \int_V \epsilon(\mathbf{r})\mathbf{E}_j \cdot \mathbf{E}_j \, dV + \frac{c^2}{2(\omega_j - i\gamma_j)^2} \oint_S \left[\mathbf{E}_j \cdot \left(\mathbf{V}_j + x\frac{\partial \mathbf{V}_j}{\partial x} + y\frac{\partial \mathbf{V}_j}{\partial y} + z\frac{\partial \mathbf{V}_j}{\partial z} \right) - r\mathbf{V}_j^2 \right] dS. \tag{6}$$

Here, $r = \sqrt{x^2+y^2+z^2}$ is the distance from origin (the center of the unit cell) to the surface of integration and $\mathbf{V}_j = (\partial \mathbf{E}_j/\partial x)(x/r) + (\partial \mathbf{E}_j/\partial y)(y/r) + (\partial \mathbf{E}_j/\partial z)(z/r)$.

Since the Green function in Eq. (4) has been defined in terms of a series of normalized eigenfunctions, it can be used to find the scattered field [16] for any source distribution. Additionally, only one dominant resonant mode, $\mathbf{E}_j$, is considered to be excited in a certain frequency range, which greatly simplifies the analytical expression for the scattered field expressed in terms of the Green function.

In the case of our nonlinear metasurface, a quasi-BIC mode $\mathbf{E}_1$ at the FF with complex frequency $\omega_1 - i\gamma_1$ dominates the scattered linear field under normally incident plane wave. At the SH, another mode, $\mathbf{E}_2$, with complex frequency $\omega_2 - i\gamma_2$ is responsible for the generated nonlinear field. Then, the total SHG power, $P_{SH}(2\omega)$, can be evaluated as follows [14]

$$P_{SH}(2\omega) = [P(\omega)\kappa_1(\omega)Q_1L_1(\omega)]^2 \kappa_{12} Q_2 L_2(2\omega)\kappa_2\alpha(2\omega). \tag{7}$$

The meaning of the parameters in Eq. (7) can be understood from the following physical considerations. The excitation source $\mathbf{E}_{bg}$ with power $P(\omega)$ couples to the quasi-BIC mode, $\mathbf{E}_1$, at the FF. The strength of the

interaction is determined by the spatial distribution of the electric-field eigenmode, $\mathbf{E}_1$, and is described by the coupling coefficient κ_1:

$$\kappa_1(\omega) = \frac{\left|\frac{\omega}{c}\int\limits_V \Delta\epsilon(\mathbf{r}',\omega)\mathbf{E}_1(\mathbf{r}')\cdot\mathbf{E}_{bg}(\mathbf{r}',\omega)\,d\mathbf{r}'\right|^2}{(2\gamma_1/c)N_1(8\pi/c)P(\omega)}. \quad (8)$$

where c is the speed of light in vacuum and the volume integral is carried out in the region where $\Delta\epsilon(\mathbf{r}',\omega)\neq 0$. Moreover, the coupling between $\mathbf{E}_1$ and $\mathbf{E}_{bg}$ is enhanced due to the large Q-factor of the quasi-BIC mode, but it can be reduced because of the spectral overlap factor, $L_1(\omega) = \gamma_1^2/[(\omega-\omega_1)^2+\gamma_1^2]$. At the quasi-BIC resonance $\omega=\omega_1$ and hence $L_1 = 1$.

The SH induced field is dominated by the eigenmode $\mathbf{E}_2$ around the SH, thus the frequency conversion efficiency depends on the second-order susceptibility tensor $\chi_{ijk}^{(2)}$ of MoS_2 layer and the spatial overlap between the interacting modes $\mathbf{E_1}$ and $\mathbf{E_2}$. This nonlinear coupling is quantified by the parameter

$$\kappa_{12} = \frac{\left|\sum_{i,j,k=1}^{3}\int \chi_{ijk}^{(2)}(\mathbf{r})E_{2,i}^*(\mathbf{r})E_{1,j}(\mathbf{r})E_{1,k}(\mathbf{r})\,d\mathbf{r}\right|^2}{(N_2\omega_2/c)(N_1\omega_1/c)^2}. \quad (9)$$

It should be noted that the nonlinearity of the optical system considered in this work originates from the single-layer MoS_2, thus simplifying the calculation of the coupling coefficient κ_{12} to a surface integral. Furthermore, the symmetry properties of the second-order nonlinearity of monolayer MoS_2 given in Eq. (2), together with the fact that the FF quasi-BIC mode $\mathbf{E}_1$ is antisymmetric with respect to reflection about the plane $x=0$, simplifies the search for a mode $\mathbf{E}_2$ around the SH. More specifically, according to Eq. (9), only eigenmodes possessing the same mirror symmetry as $\mathbf{E}_1$ can be excited.

The next step is to evaluate the out-coupling coefficient at SH, κ_2. Since the SHG intensity depends on both the quality factor Q_2 of the SH mode and the spectral overlap $L_2(2\omega_1) = \gamma_2^2/[(2\omega_1-\omega_2)^2+\gamma_2^2]$ between the modes $\mathbf{E_1}$ and $\mathbf{E}_2$, we include their effects in the evaluation of SHG in Eq. (7). In the case when $2\omega_1 = \omega_2$, the frequency overlap of FF and SH modes reaches its maximum. To compute the outgoing SH power, the out-coupling coefficient κ_2 relating to SH mode $\mathbf{E}_2$ is defined:

$$\kappa_2 = \frac{\oint\limits_S \mathrm{Re}[\mathbf{E}_2\times\mathbf{H}_2^*]\cdot d\mathbf{S}}{(2\gamma_2/c)N_2}. \quad (10)$$

Here, the surface integral is evaluated in two cross-sections normal onto the z-axis, one located in the upper free-space region and the other one in the quartz substrate. Finally, the parameter $\alpha(2\omega) = (8\pi/c)(2\omega/c)^2$ in Eq. (7) is used as a smoothing factor.

Given the above discussion, the SHG intensity from a monolayer MoS_2 deposited on the cruciform-shaped silicon metasurface, whose specific expression is given in Eq. (7), can be determined by combining Eq. (8), (9), and (10), together with the Q-factors Q_j and spectral overlap factors L_j. This semi-analytical method provides a powerful quantitative tool to understand and quantify each step in the frequency conversion process.

6 Nonlinear homogenization method

In this section, we present a versatile nonlinear homogenization method [17, 18] used to quantitatively study the underlying physics of the nonlinearity enhancement in our nonlinear metasurface. Based on the second-order susceptibility of monolayer MoS_2 and the FF near-field distribution, the nonlinear polarization at SH frequency is defined as

$$\mathbf{P}^{nl}(\mathbf{r},2\omega) = \epsilon_0\boldsymbol{\chi}^{(2)}(\mathbf{r},2\omega,\omega,\omega):\mathbf{E}(\mathbf{r},\omega)\mathbf{E}(\mathbf{r},\omega), \quad (11)$$

where $\boldsymbol{\chi}^{(2)}$ is the second-order susceptibility with nonzero values only in the region of monolayer MoS_2. Due to the tensor properties of the second-order polarization, its components can be written as

$$P_i^{nl} = \epsilon_0\sum_{jk}\chi_{ijk}^{(2)}E_jE_k \equiv \epsilon_0\sum_{jk}q_{ijk}. \quad (12)$$

Here, the auxiliary physical quantities $q_{ijk} = \chi_{ijk}^{(2)}E_jE_k$ are introduced and their spatial average is defined as

$$\overline{q}_{ijk}(2\omega) = \frac{1}{V}\int\limits_V \chi_{ijk}^{(2)}(\mathbf{r},2\omega,\omega,\omega)E_j(\mathbf{r},\omega)E_k(\mathbf{r},\omega)\,d\mathbf{r}. \quad (13)$$

In addition to Eq. (12), the second-order nonlinear polarization can also be expressed using an effective second-order susceptibility, $\boldsymbol{\chi}_{\text{eff}}^{(2)}$, and averaged electric fields $\overline{\mathbf{E}}(\omega) = \left(\int_V \mathbf{E}(\mathbf{r},\omega)\,d\mathbf{r}\right)/V$ at FF, where V is the integral volume (monolayer MoS_2), as

$$\overline{P}_i^{nl}(2\omega) = \epsilon_0\sum_{jk}\chi_{\text{eff},ijk}^{(2)}(2\omega,\omega)\overline{E}_j(\omega)\overline{E}_k(\omega). \quad (14)$$

To guarantee equality of the averaged nonlinear optical response between the cruciform-shaped MoS_2 monolayer and the homogenized layer, one requires that the expressions of nonlinear polarizations in Eq. (12) and (14) are *termwise* identical. Therefore, the effective second-order susceptibility $\chi^{(2)}_{\text{eff}}$ can be calculated as

$$\overline{\chi}^{(2)}_{\text{eff},ijk}(2\omega,\omega,\omega) = \frac{\overline{q}_{ijk}(2\omega)}{\overline{E}_j(\omega)\overline{E}_k(\omega)}. \qquad (15)$$

References

[1] Synopsys' DiffractMOD, https://www.synopsys.com.
[2] K. Levenberg, "A method for the solution of certain problems in least squares," *Quart. Appl. Math.*, vol. 2, no. 2, pp. 164–168, 1944.
[3] D. W. Marquardt, "An algorithm for least-squares estimation of nonlinear parameters," *SIAM J. Appl. Math.*, vol. 11, no. 2, pp. 431–441, 1963.
[4] COMSOL Multiphysics, https://www.comsol.com.
[5] CST Studio Suite, https://www.3ds.com/products/simulia/cst-studio-suite.
[6] J. W. You, E. Threlfall, D. F. G. Gallagher, and N. C. Panoiu, "Computational analysis of dispersive and nonlinear 2D materials by using a GS-FDTD method," *J. Opt. Soc. Am. B*, vol. 35, no. 11, pp. 2754–2763, 2018.
[7] C. Schinke, *et al.*, "Uncertainty analysis for the coefficient of band-to-band absorption of crystalline silicon," *AIP Adv.*, vol. 5, no. 6, p. 067168, 2015.
[8] L. V. de Rodríguez-Marcos, J. I. Larruquert, J. A. Méndez, and J. A. Aznárez, "Self-consistent optical constants of SiO_2 and Ta_2O_5 films," *Opt. Mater. Express*, vol. 6, no. 11, pp. 3622–3637, 2016.
[9] Y. Li, *et al.*, "Measurement of the optical dielectric function of monolayer transition-metal dichalcogenides: MoS_2, $MoSe_2$, WS_2, and WSe_2," *Phys. Rev. B*, vol. 90, no. 20, p. 205422, 2014.
[10] M. Weismann and N. C. Panoiu, "Theoretical and computational analysis of second- and third-harmonic generation in periodically patterned graphene and transition-metal dichalcogenide monolayers," *Phys. Rev. B*, vol. 94, no. 3, p. 035435, 2016.
[11] L. M. Malard, T. V. Alencar, A. P. M. Barboza, K. F. Mak, and A. M. de Paula, "Observation of intense second harmonic generation from MoS_2 atomic crystals," *Phys. Rev. B*, vol. 87, no. 20, p. 201401, 2013.
[12] B. Zhen, C. W. Hsu, L. Lu, A. D. Stone, and M. Solja, "Topological nature of optical bound states in the continuum," *Phys. Rev. Lett.*, vol. 113, no. 25, p. 257401, 2014.
[13] T. Yoda and M. Notomi, "Generation and annihilation of topologically protected bound states in the continuum and circularly polarized states by symmetry breaking," *Phys. Rev. Lett.*, vol. 125, no. 5, p. 053902, 2020.
[14] K. Koshelev, *et al.*, "Subwavelength dielectric resonators for nonlinear nanophotonics," *Science*, vol. 367, no. 6475, pp. 288–292, 2020.
[15] M. B. Doost, W. Langbein, and E. A. Muljarov, "Resonant-state expansion applied to three-dimensional open optical systems," *Phys. Rev. A*, vol. 90, no. 1, p. 013834, 2014.
[16] F. Alpeggiani, N. Parappurath, E. Verhagen, and L. Kuipers, "Quasinormal-mode expansion of the scattering matrix," *Phys. Rev. X*, vol. 7, no. 2, p. 021035, 2017.
[17] Q. Ren, J. W. You, and N. C. Panoiu, "Large enhancement of the effective second-order nonlinearity in graphene metasurfaces," *Phys. Rev. B*, vol. 99, no. 20, p. 205404, 2019.
[18] J. W. You and N. C. Panoiu, "Plasmon-induced nonlinearity enhancement and homogenization of graphene metasurfaces," *Opt. Lett.*, vol. 44, no. 12, pp. 3030–3033, 2019.
